# A Qualitative Study on the Experiences of Adult Females with Late Diagnosis of ASD and ADHD in the UK

**DOI:** 10.3390/healthcare14020209

**Published:** 2026-01-14

**Authors:** Victoria Wills, Rhyddhi Chakraborty

**Affiliations:** 1 Registered Mental Health Nurse, Priory Hospital Norwich, Ellingham Road, Attleborough NR17 1AE, UK; vewills26@gmail.com; 2 Faculty of Health, Medicine and Social Care, School of Allied Health and Social Care, Anglia Ruskin University, Bishop Hall Lane, Chelmsford CM1 1SQ, UK

**Keywords:** ASD, ADHD, adult females, diagnosis, healthcare professional

## Abstract

**Background:** Adult females with Autism Spectrum Disorder (ASD) and Attention Deficit Hyperactivity Disorder (ADHD) are frequently underdiagnosed due to gender bias, overlapping symptoms, and limited awareness among healthcare professionals. The scarcity of research on this subject—particularly in the UK context—underscores the need for further investigation. Accordingly, the aim of this study was to explore the lived experiences of adult females receiving a late diagnosis of ASD and/or ADHD and to identify key barriers within the UK diagnostic pathway. This study addresses a critical knowledge gap by examining the factors contributing to delayed diagnosis within the United Kingdom. **Study Design and Method:** The study employed a qualitative approach, utilising an anonymous online questionnaire survey comprising nine open-ended questions. Responses were obtained from 52 UK-based females aged 35–65 years who had either received or were awaiting a diagnosis of ASD and/or ADHD. Data were analysed thematically within a constructivist framework. **Findings:** The analysis revealed three overarching themes: (i) limited understanding and lack of empathy among healthcare professionals, (ii) insufficient post-diagnostic support, with most participants reporting no follow-up care, and (iii) a complex, protracted diagnostic process, often involving waiting periods exceeding three years. Gender bias and frequent misdiagnosis were recurrent issues, contributing to significant psychological distress. These findings underscore the need for systemic reforms and align closely with gaps identified in the existing literature. **Conclusions:** The findings emphasise the urgent need for gender-sensitive diagnostic frameworks, enhanced professional training, and a person-centred approach to care. Key recommendations include shortening diagnostic waiting times, strengthening healthcare professionals’ knowledge base, and ensuring equitable and consistent post-diagnostic support.

## 1. Introduction

Autism Spectrum Disorder (ASD) and Attention Deficit Hyperactivity Disorder (ADHD) are two different neurodevelopmental disorders that impact people differently. While autism is distinguished by limited and repetitive activities and interests, as well as challenges with social communication and engagement, ADHD is characterised by symptoms of inattention, hyperactivity, and impulsivity. Despite these differences, it can be challenging to distinguish between the two since symptoms including problems with self-control, concentration, and social interactions may overlap [1,2], and 50–70% of individuals suffer from the double burden of ASD and ADHD.

The literature suggests that ASD and ADHD are difficult to diagnose and distinguish [3] and also reports increased rates of comorbidity and significant symptomatic overlaps between Bipolar Disorder (BPD) and ASD symptoms and traits in adults [4,5,6]. Hence, some persons may have co-occurring diseases that make it challenging to diagnose and distinguish if the symptom is of ASD or ADHD. Additionally, some mental health conditions might be present with symptoms including features of both [3,7]. Research has also demonstrated that, consequently, many adult individuals will be misdiagnosed or undiagnosed for ASD and/or ADHD and would frequently obtain a variety of differential psychiatric diagnoses before receiving the proper neurodevelopmental diagnosis for ASD and/or ADHD [8,9]. The overall rate of diagnoses for all individuals with ADHD or ASD, in the United Kingdom (UK), has risen from 0.5% in 2017 to 1.2% in 2023 [10]; however, there is a dearth of enhanced study into the assessment and diagnostic process, particularly in adults. ADHD in adults differs from ADHD in children in appearance and behavioural patterns. The accuracy of the evaluations is compromised since the diagnostic criteria employed by clinicians, such as age cutoffs and symptom wording, are not differentiated for adults. The use of unclear clinical criteria makes diagnosing ASD and ADHD in adults even more difficult. Clinical recommendations employ the Diagnostic and Statistical Manual of Mental Disorders, 5th edition (DSM-5) criteria, which are based on the International Statistical Classification of Diseases and Related Health Problems (ICD) but are not very detailed [11,12,13,14]. The DSM-5 is very much an open and malleable tool, rather than a checklist, and, as such, depends entirely on correct, consistent, trained gatekeepers and diagnostic specialists [15]. It has been noted that the DSM-5 added ASD as a construct with an unclear identity and no well-defined diagnostic criteria, which presented extra difficulties for parents and practitioners. At the same time, each ASD condition continues to have its own distinct characteristic requiring precise diagnosis [13]. Studies found that diagnostic and screening tools for both ASD and ADHD, are laden with criteria that also emphasises the male presentation, developed using male samples and potentially exclude many female dominant traits and symptoms [7,16,17]. The male-predominant prevalence ratios may have resulted in longstanding male-centred clinical prototypes in clinicians’ minds as well as under-diagnosis in women [17]. Additionally, there is a long-standing belief that ASD and ADHD are relatively uncommon in women because of their less outward behaviours [18,19]. Because of anatomical similarities and limited awareness, healthcare professionals often misinterpret female ASD/ADHD symptoms as mental health conditions such as depression, anxiety, and eating disorders [3,19], often leading to a late diagnosis of ASD/ADHD. It is believed that 50–75% of females are undiagnosed with ADHD, and nearly 80% ASD females have been previously misdiagnosed [20,21]. Hence, this study investigates factors contributing to delayed diagnosis of ADHD and ASD in adult females in the UK. Due to their high comorbidity and significant overlap in symptoms, ASD and ADHD were combined in this study [3,22]. ADHD frequently co-occurs with ASD, significantly more than in individuals without ASD. To improve treatment outcomes, healthcare facilities should implement robust systems for tracking and assessing ADHD comorbidity in the ASD cases and ensure more accurate diagnostic processes for these disorders [22]. Research also indicates that the provision of healthcare, in this context, is conditioned by the healthcare workers’ knowledge, understanding, and attitude to engage with persons with ASD/ADHD [23]. Healthcare professionals’ understanding of these conditions, along with ensuring equitable support for individuals with ASD and ADHD, can be strengthened through further research in this area. To fill the explicit gaps in provision and practice, the study was undertaken with the special focus on ASD and/or ADHD of females in the UK.

## 2. Study Design and Methodology

The study aimed to compare the experiences of adult females who received a late diagnosis when discussing ASD and/or ADHD with healthcare professionals in the UK. To achieve this aim, the study was guided by three specific objectives: (i) to evaluate the perspectives of late-diagnosed adult females regarding their initial interactions with healthcare professionals during the diagnosis-seeking process; (ii) to assess the extent to which healthcare professionals are aware of and comprehend ASD and/or ADHD in adult females, as reflected in participants’ perspectives; (iii) to evaluate participants’ perceptions of the depth and accuracy of healthcare professionals’ knowledge concerning ASD and/or ADHD. In order to achieve these aims and objectives, the study adopted a primary qualitative research design informed by the constructivist paradigm. A primary study was chosen to gain information directly from those who have experience in this area. Using a qualitative method of analysis allowed the participant responses to be mapped and compared, providing transparency with identifiable themes. The constructivist paradigm was selected to reflect the study’s emphasis on comprehending participants’ subjective perspectives and interpretations of their experiences [24,25].

This has been used to also ensure understanding of the real-life experiences of the participants’ progress through the assessment and diagnostic processes for ASD and/or ADHD [26,27]. A deductive research approach was adopted, reflecting the growing scholarly and clinical concerns surrounding this topic. Both authors conducted a comprehensive review of existing studies and theoretical frameworks, subsequently re-examining and assessing these through a qualitative design to refine current understandings and generate further insight [27,28].

To improve the contextual knowledge and to gather the opinions and perspectives of the participants, phenomenological components were added, and all processes adopted ethical guidelines that ensured the objectivity and integrity of the results. The SPIDER (Sample, Phenomenon of Interest, Design, Evaluation, Research type) conceptual framework was used to create interview questions, which helped the authors to align the research question with the objectives of the study.

## 3. Data Collection

This study included adult females residing in the UK, between 35 and 65 years of age, who had either received a diagnosis of ASD and/or ADHD; or were awaiting diagnosis. The study excluded males, females under 35 or over 65 years of age, and individuals residing outside the UK. The research focused on participants’ experiences of engaging with healthcare professionals and the experience of the overall diagnostic process. A purposive sampling strategy was employed to ensure that data analysis remained closely aligned with the research objectives, thereby enabling a comprehensive exploration of the subject area [28,29,30]. In accordance with DSM-5 criteria and with NHS approval, participants were selected from the UK target population and assessed by ASD and ADHD specialists through clinical interviews, behavioural observations, and corroborative data [31,32,33]. The study was initially designed for a sample size of 50 participants; however, upon completion, 52 responses were received, of which 6 did not meet the inclusion criteria. Data collection was originally scheduled from 18 May 2024 to 14 June 2024, but due to the rapid response rate, the questionnaire was closed early on 23 May 2024. After 50 survey responses, the phenomenological data saturation was reached since further replies from the targeted demographic did not reveal any new themes [34]. This qualitative study employed an open-ended questionnaire as the primary data collection tool. The instrument included three checkbox items addressing consent, age bracket, and diagnostic status, followed by nine open-ended questions designed to elicit detailed, subjective responses from participants. The open-ended questions sought information about participants’ experiences with healthcare professionals, spanning the initial referral discussion through to post-diagnostic interactions. These questions also explored how professionals responded to participants’ reasons for requesting a diagnosis and whether additional support was offered following diagnosis. The final question invited participants to provide any further comments on the topic.

The online survey platform at Anglia Ruskin University was used to create the questionnaire, which was then shared on public social media platforms including Facebook and LinkedIn. Participants, living in the UK, were recruited using open and public groups on these platforms, particularly those that cater to people with ASD and ADHD. The questionnaire was created in a way that prevented participants from answering or submitting answers without first verifying their consent, in order to guarantee informed consent. This method ensured that participants were aware of the specifics of the study before they took part. An advertisement was made to promote the research specifics, including links to the participant information sheet and the university’s and the survey application’s privacy rules. Additionally, a QR code and embedded links led participants to the actual questionnaire. The survey was filled out in an anonymous manner.

The authors collaborated closely during the preparatory phase and conducted a comprehensive literature review to strengthen conceptual grounding. Questionnaire content was aligned with the study aim and objectives, and participants were recruited through appropriate channels to ensure relevance and representativeness. The findings were consistent with prior research and reflective of the targeted population.

## 4. Data Analysis

The data were analysed thematically within a constructivist paradigm. Participant narratives were instrumental in developing a nuanced understanding of the context and organising insights into coherent themes [35].

This approach enabled a descriptive and subjective interpretation of the lived experiences of participants, thereby capturing the complexity and depth of these experiences [36,37].

**Figure 1:**
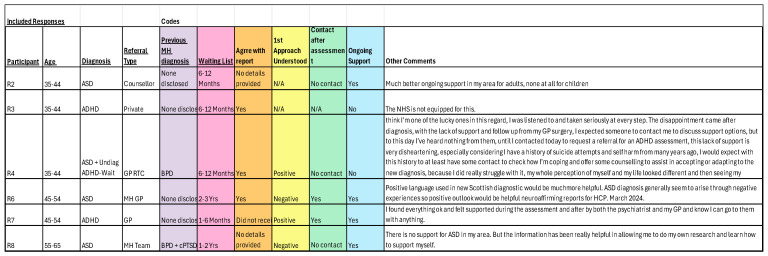
Snapshot of organisation tool.

The anonymised data was collected and allowed simple organisation, separating the information by each question. A spreadsheet was created with all participant responses, using filters to identify variables. Figure 1 shows how this was organised, with each category visible with the option to unhide the comments made, for full analysis.

**Figure 2:**
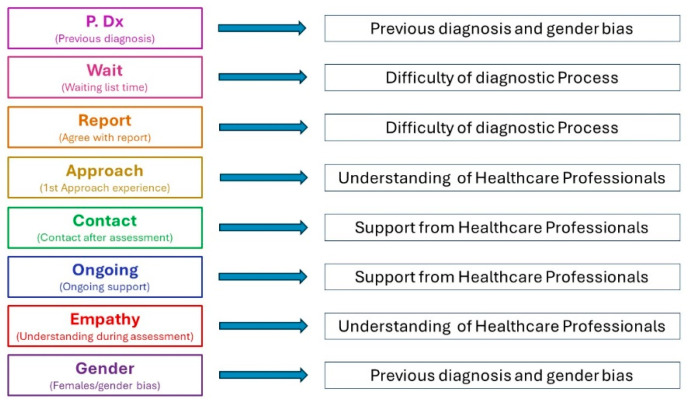
Codes into themes.

**Figure 3:**
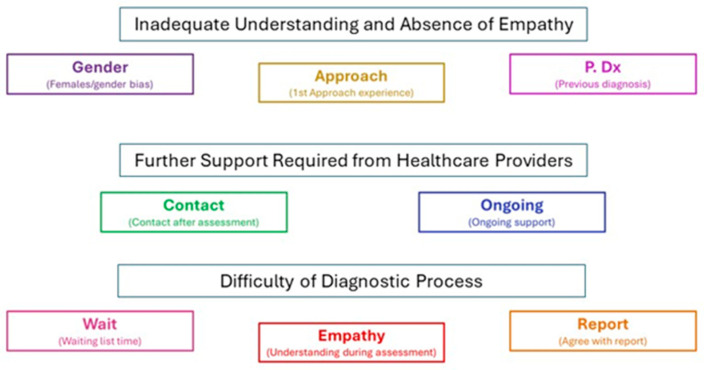
Summary of the final themes.

Organising the information in this way allowed initial codes to be generated by the information provided and helped to conduct a thematic analysis [38]. Initial codes and themes were found to be linked, with previous diagnosis, gender, and empathy influencing healthcare professionals’ understanding and diagnostic difficulties. Within the additional comments received from the participants, many discussed their experiences during the assessment itself. This provided the details to complete a thematic analysis to meet the research aim and objectives. By reading these additional comments, further codes were identified, resulting in eight initial codes to begin the analysis process (Figure 2). Examining the information in the coded areas, three main themes were finally identified (Figure 3). These were inadequate understanding and absence of empathy, requirement of further support from healthcare providers, and difficulty in the diagnostic process.

The research, coding, and thematic analysis were completed by the first author, and validation and supervision of research was conducted by the second author. Since the research aim and objectives served as the foundation for the study, going back to the initial research objectives helped define the research themes. To find the best approach to comprehend the data and the relationships between each category, the information was carefully examined. Descriptive titles were then assigned to the themes. Each of the research objectives relate back to the codes and themes identified, as the data has provided the experiences and perspectives of adult females with ASD and/or ADHD, or those awaiting diagnosis.

The process of analysing data in this way, has provided insight into those experiences. Adhering to thematic analysis guidelines facilitated a robust representation of the phenomenon under study and ensured an accurate reflection of the target population. The overall findings generated recommendations aimed at enhancing education, clinical practice, and future research in this area.

## 5. Validity and Reliability

Ensuring validity and reliability is fundamental in designing, organising, and monitoring qualitative research to establish credibility. A study is considered valid when it accurately represents the variables under investigation and is reliable when its findings are consistent with similar research [39,40,41,42]. In this study, validity was supported through anonymous review of the questionnaire items to confirm their alignment with the original concepts, reflecting and disseminating the exact experiences shared in the survey. In order to have the precise presentation and to ensure that the analysis was in compliance with the research design and objectives, direct quotes were also used. Reliability was enhanced through the use of a standardised questionnaire and providing information of the research study to the participants in advance, thereby, gaining trustworthiness and credibility. The information shared in advance outlined the title, purpose, and requirements of the study, information regarding ethical approval, permissions and legislation related to the study, and dissemination of the outcome of the study. The contact details of the authors and the university complaints department were also provided. Further specific information was also provided on data collection, storage, and duration of data storage, through the requirements of data protection, information security, and records retentions policies [43]. Additionally, the reliability was maintained in representing the exact lived experiences of the participants through interpretations with direct quotes and analysis.

Responses were collected anonymously via the university’s online survey platform, with no identifying details recorded. The authors maintained no personal relationship with any participants, thereby, minimising potential bias. All responses adhered to the criteria outlined in the Participant Information Sheet. Data were analysed anonymously and presented transparently using participants’ verbatim comments. Study limitations are acknowledged in a later section, and findings were compared with the literature review to ensure consistency, reliability, and trustworthiness.

Ethical Considerations: The ethical approval was granted by the School of Research Ethics Panel (SREP), Faculty of Health, Medicine and Social Care (HEMS), Anglia Ruskin University (ARU). The study was of medium risk due to the use of human participants. This required a risk assessment to ensure steps were taken to minimise these potential risks. The risks identified were mitigated by the following steps: not collecting personal information; providing advice and information for mental health support to participants; securely storing all data in the university’s secure data drive, accessible by the authors only; not recruiting known participants to the authors to remove bias; considering consent before data collection prior to submission of the questionnaire responses. Throughout the course of the research, this study adhered to all ethical standards, ensuring the validity, accuracy, and authenticity of the data gathered and their interpretation.

## 6. Findings

Of the 52 responses received, 46 met the inclusion criteria set out during the planning of the study. The six responses were excluded for not meeting the inclusion criteria and were from females between 18 and 34 years of age.

Female participants in the 35–44 age range had the highest response rate, while those in the 55–65 age range had the lowest.

The study involved 23 participants with ADHD, 8 with ASD, 7 with a dual diagnosis, 11 awaiting further diagnosis for the additional disorder, and 8 awaiting diagnosis for one or both. The study involved 19 participants who disclosed a previous mental health diagnosis, with 12 disagreeing and 3 not stating their agreement, leaving 4 participants agreeing with their diagnosis. Participants were assigned numerical identifiers in sequential order based on their response submission.

A total of 33 participants were referred to a diagnostic service through their General Practitioner, either through the NHS England or the Right to Choose pathway, allowing patients to fully participate in their healthcare and treatment options, as per the NHS Constitution for England [44,45]. However, as the study identified, gaps exist in practice. These findings underscore the need for systemic reforms in provisions and practice and align closely with gaps identified in the existing literature.

Theme 1: Inadequate Understanding and Absence of Empathy

The theme was influenced by comments on referral approach, previous diagnosis, gender bias, and assessment experience.

The study revealed that participants’ interactions with healthcare professionals were fairly balanced, with 16 participants reporting positive experiences and 15 participants reporting negative ones.

Three participants reported changing providers after a negative experience, and both positive and negative experiences were evenly distributed between ASD and ADHD.

Positive interactions that were shared, reported supportive healthcare professionals as in the responses from R4 and R38 (Table 1).

Less information was provided by participants with positive experiences. Although, the same use of words followed through the responses, including “My GP made me feel validated”.

Participants discussing a negative experience expressed frustration from these initial interactions. These feelings were echoed in response from R40, for instance (Table 2).

Other than positive and negative experiences of the first interaction, participants also expressed their experiences and consequent feeling from misdiagnosis and lack of diagnosis. The quote from R13 reflects such experience. Four participants had agreed with their previous diagnosis of a mental health condition; however, twelve participants disagreed with their previous diagnosis of a mental health condition and often related their symptoms to undiagnosed ASD or ADHD. Two participants mentioned continuing with the treatment plan stated to them in the previous diagnosis (Table 3).

Within the additional comments, six participants remarked on gender bias, implied a lack of empathy, and expressed the struggles they had endured from this throughout their journey, from the initial consultation to post-diagnosis. Quotes from R9 and R25 reflect such remarks (Table 4).

Theme 2: Further Support Required from Healthcare Providers

The theme was generated from the participants’ comments on whether their healthcare professionals contacted them post-assessment and whether they received any ongoing support.

Out of 31 responses received on post-assessment follow-up, 9 responses confirmed being contacted by their healthcare professionals, leaving 22 without contact. Quotes from R26, R28, and R38 reflect those participants who were contacted by their healthcare professionals, and discussed support options, history of symptoms, self-care, medical history, and related health issues (Table 5).

Quotes from R4 and R40 reflect the experience of no response from healthcare professionals (Table 6).

On the context of ongoing support, a total of 34 responses were received; only 6 participants reported receiving support, meaning 28 did not receive ongoing assistance from either their primary or secondary healthcare provider.

Of the 28 participants who reported that no further support was offered, 21 commented on this topic, with 7 participants stating they had only been offered medication. Participant R48 noted that, despite being offered medication titration and therapy, they are still waiting for further follow-up. Some other participants stated that even though ongoing support was provided, it was often very limited in timescale, as reflected in the following quotes (Table 7).

Some participants shared they had been offered shared care for medication after diagnosis by a private diagnostic service, but no direct support was provided. Participant R39 reported “Post diagnostic support group and literature from trust” highlighting that although the primary healthcare provider did not offer support, the local NHS trust of this participant did provide support to the participant.

Theme 3: Difficulty of Diagnostic Process

Participants’ comments on waiting list length, agreement with diagnosis report, and empathy and understanding of healthcare professionals were considered for this theme.

The study involved 46 participants who provided a timeframe for their wait between initial referral and assessment, and those still waiting indicated the expected timeframe.

There were 19 participants who waited for less than a year for assessment, and many had been referred by their GP through the Right to Choose pathway [39]. Of these, six participants waited less than 6 months, three referred themselves directly to a private diagnostic service. Of the 27 participants who waited for over a year, 10 reported having waited for more than 3 years, and 1 participant (R28) was even hinted to have a waiting time of 8 years (Table 8).

When asked if they agreed with their diagnostic report, 24 of the participants commented on this. There were eight participants who agreed with the details provided, five and participants stated non-receipt of the report or that specific details were not included. Those who disagreed with the details included in their report provided comments (Table 9).

Of the participants who agreed with their diagnostic reports, three stated that they felt it was an accurate diagnosis (Table 10).

The category of empathy from healthcare professionals was constructed through the additional comments where participants expressed not just inadequate support but also disappointment with the assessment process (Table 11).

The six participants excluded from the study reported much the same experiences as those who were included.

## 7. Discussion

The findings indicate that many females were diagnosed late due to the difficulty of the diagnostic process and also highlight the provision of diagnosis and treatment made by the healthcare professionals they interacted with. The data analysis showed clear patterns which guided the creation of themes for additional analysis. Furthermore, these findings demonstrate congruence with the existing literature and prior empirical studies on this topic [46].

The first theme of the study looked at the understanding and empathy shown by healthcare professionals. Many of the participants reported negative experiences on their initial approach to their healthcare professional. They reported having their concerns dismissed, leaving them feeling frustrated, angry, and unsupported. This question elicited many negative emotions within the answers from the participants.

This has been seen in previous studies, where research participants felt that their healthcare professional did not understand their concerns or hold comprehensive knowledge of these disorders [6,7,47]. With other studies remarking that there is often a lack of awareness of these conditions amongst clinicians [6,7,45]. The study highlights the need for increased education on neurodevelopmental disorders, particularly in general practice primary care settings, as many of the participants believed they had been misdiagnosed or their symptoms misunderstood at this stage, leading to increased anxiety and depression.

Of those participants who disclosed a previous diagnosis for a mental health condition, the majority felt that this was a misdiagnosis or a misunderstanding of symptoms of undiagnosed ASD and/or ADHD. This was particularly noticeable in those diagnosed with anxiety and depression. Misdiagnosis is often caused by the overlap in symptoms with a number of mental health conditions. Previous studies also found that these were the most commonly misdiagnosed conditions. Individuals with ASD and/or ADHD were found to be at a higher risk of developing anxiety and depression, with elevated feelings of suicidality [5,6,20,46,47]. This confusion and lack of awareness by healthcare professionals also contributed to late diagnosis in many. This information further proves the need for education and improved knowledge amongst clinicians, and a need for more specialist services.

Some research participants discussed concerns around gender bias, feeling they were not listened to or dismissed by their healthcare professionals. These comments question the knowledge held by healthcare professionals around the female phenotypical presentation of ASD and ADHD. This is also a concern of published researchers on this topic [48,49]. Statements include that some clinicians overlook symptoms in females due to less overt symptoms, as these are more likely to be manifested internally [8,18,20].

There is also the concern that the screening tools for ASD and ADHD impact on gender bias in diagnosis and treatment. Screening tools for ASD and ADHD have historically been developed and standardised using predominantly male samples, which limits their applicability to females. This lack of gender-sensitive diagnostic instruments, combined with insufficient understanding of gender-based differences, continues to contribute to underdiagnosis amongst women [5,23].

The study underscores a significant gap in the current knowledge, with the need of further research to highlight gender differences presented within these disorders. Improvements to the current diagnostic tools are also required, with further guidance on its appropriate use.

These results meet the research objectives in a number of ways. The participants’ perspective of initial engagement and the knowledge held by their healthcare professional have been compared and evaluated. The level of awareness and understanding held by healthcare professionals has also been evaluated and discussed, as seen from the participants’ perspective. From these results and a review of the wider literature, ASD and ADHD in females appears to require healthcare professionals to increase their knowledge and understanding of these disorders.

The second theme identified found that further support is required from healthcare providers. The majority of research participants reported that they received no contact from the healthcare professional after assessment. They also reported that ongoing support was not offered. Some participants stated that recommendations of support were provided to their primary healthcare professional, but a follow up discussion about these did not take place. Of those that were contacted, with an ADHD diagnosis, only medication was offered. Those that were assessed through a private diagnostic service were offered a shared care agreement to manage medication. Of the group that were offered support, most were advised to self-refer to external organisations.

This information has provided insight into the services available to those diagnosed with ASD and/or ADHD. Therapeutic services for neurodevelopmental disorders in the UK often fall under the remit of local mental health teams [48,49]. Over the past few years, it has been widely reported that there is a lack of availability and long waiting lists for support within the NHS [50,51]. On this subject, there is also a lack of research available. However, there is mention of the need to improve services and ongoing support, with recommendations for improvement of continuation of care [18,45,52]. The National Institute for Health and Care Excellence (NICE) guidelines recommend that support is to be provided to all individuals after diagnosis [53].

Although there are specialised services available, it appears that there is not enough provision to support the number of individuals that are diagnosed. This study has proved that investment is required, in the form of clinicians and support programmes, to provide the much-needed equitable assistance.

The last theme identified from the results achieved was regarding the difficulty of the diagnostic process. All of the participants that responded were informed of the wait time between referral and assessment. Over half of these individuals waited over a year for assessment, with many waiting over 3 years. The participants that spent this extended period of time to be assessed were referred through the NHS diagnostic pathway.

NHS England [54] states within their clinical guidelines that individuals should be seen within 3 months for ASD assessment, but current demand exceeds capacity. There is no recommended timeframe listed for ADHD, but they have recognised that a timely assessment is vital [43]. A recent study has shown a rapid increase in demand for ASD and ADHD assessments, which has severely impacted the length of time patients wait for assessment [32,53].

There is a clear need for increased availabilities and accessibilities. The study highlights the need for an improved assessment infrastructure, as individuals often face difficulties due to wait times [55]. Most participants felt that the healthcare professional’s report did not cover all areas or accurately recognise the severity of symptoms. Individuals requiring assessment have stated the difficulties they experience because of the wait times. Without diagnosis and support, individuals will continue to struggle in managing their day-to-day lives. These also link back to the literature which shows that adult females are less likely to be diagnosed correctly, often due to presenting with less overt symptoms, overlap of symptoms between disorders, and the need for improved diagnostic tools to include the female phenotype [5,8,19].

The last contributing factor to this theme is the empathy expressed by clinicians during the assessment. The participants discussed feelings of disappointment and lack of care, with some stating this process was challenging and traumatic. Some stated that their clinician was rude, dismissive, and unsupportive. The study, therefore, emphasises the need for improved written communication and inclusion of relevant details in assessment reports, as well as the need for improved empathy from healthcare professionals, who often displayed a lack of care and an unsupportive approach. A number of study participants discussed their thoughts on the diagnostic report they received. A small amount of these received a detailed report, most of which did not agree with the details included. They felt that the assessing clinician either did not cover all areas, or that their interpretation did not recognise the severity in which the individual experienced their symptoms.

Previous studies have not been found that express participants’ views on assessing healthcare professionals. This information does, however, correlate with recommendations from the wider literature regarding the lack of awareness and lack of appropriate communication with patients. Also, a thorough assessment by healthcare professionals experienced in diagnosing the female population is recommended [6,18,44].

This study has shown that individuals accessing these services often find the assessment experience distressing. Further care and understanding are required, with a focus on individualised person-centred care.

This information meets the research objectives by comparing the perception of knowledge held about ASD and ADHD by clinicians. It also gives the participants perspectives of the level of awareness and understanding that their healthcare professional holds. This further supports the overall aim of research, as it compares various aspects of the experiences that adult females engage in to receive their diagnosis.

Although not a definitive answer to the question of why adult females are often late diagnosed with ASD and ADHD, these results show many of the contributing factors and barriers to diagnosis. The study participants evidenced their negative experiences with healthcare professionals and the difficulty they have experienced with the diagnostic process. It has shown a lack of understanding and empathy, at times; and, in some cases, a misunderstanding of symptomology. Practical barriers to diagnosis have also been discussed, with wait times and support options.

## 8. Limitations

This study confirms existing evidence relating to the late and misdiagnosis of adult females with ASD and/or ADHD. It also gives further evidence of the need for increased knowledge by clinicians, with ongoing support required for better patient outcomes. The data provides additional insight into the patient experience when engaging with healthcare services and contributes to existing research by viewing the process of diagnosis and any aftercare that is provided. However, the study’s scope is limited, as the final sample comprised 46 participants. Further research, with a larger sample is warranted, given the current prevalence of ASD in UK adults (1.1%) and ADHD (3–4%), providing meaningful insights into the research problem [10,11].

Additionally, due to participants’ anonymities and recruitment via social media platforms, and by targeting a specific demographic characteristic, some degree of selection bias might be present. Future research could address this limitation by verifying the responses through face-to-face interaction, leaving certain confidential details anonymous. Furthermore, even though all participants were adults at the time of diagnosis, it is still unclear how recent the diagnosis was because the data collecting instrument did not record the precise age of diagnosis. Recording this specific information in the future can address the limitation.

The study’s omission of healthcare experts’ viewpoints, which, when combined with patient viewpoints, would offer a more thorough knowledge of the problem, is a further limitation. A more thorough understanding of the provider and receiver of services may be obtained by more research on the matching of thoughts and perspectives.

Because the sample was selected from an appropriate demographic and all replies are given strictly in the participants’ own words, the results are nonetheless reliable despite these constraints. Additionally, all participants were citizens of the United Kingdom, guaranteeing that diagnoses followed standardised screening and diagnostic processes authorised by the National Health Service.

## 9. Recommendations

The study suggests more research on patient experiences and healthcare professionals’ perspectives on ASD and ADHD; improvements in healthcare professionals’ education and awareness; improved screening tools to guarantee correct administration of assessments and easy identification of all symptoms that may be present within these disorders; a more seamless transition from referral to assessment; and better communication between the providers and recipients. Enhanced support enables treatment choices and better health outcomes. Reduction in waiting lists may all be achieved by improving healthcare delivery, adequate knowledge, and enhanced communication. Individualised care, better service management, and enhanced reputation can all be fostered in a care setting. The study emphasises that healthcare services will be better able to satisfy the needs of their clients if they offer person-centred care and treatment with better health outcomes.

## 10. Conclusions

This research study analysed the experiences and perspectives of adult females in the UK, late diagnosed with ASD and/or ADHD. The information received was in relation to the process of referral, assessment and after care, and the interactions with their healthcare professionals. Through analysing this primary qualitative study, themes were defined. Much of the information related to dissatisfaction with healthcare professionals and the barriers that are present when pursuing the correct diagnosis and treatment options.

This study has emphasised the concerns and problems faced by persons with ASD/ADHD through the diagnostic process and those currently awaiting assessment. The participants of this study expressed both positive and negative experiences with their healthcare professionals. Some participants had a positive experience upon initial meeting for referral to assessment, but the majority reported that no ongoing support had been offered post-diagnosis by their primary healthcare provider.

Limitations were found, but the validity of the study remains. By researching this area of interest, a number of recommendations have been exposed. The information has revealed a need for further research into patient experiences. Not only to confirm the data already received, but a larger study would be more representative of the target population. Research into the views and opinions of healthcare professionals would also provide a clearer picture of the problem as a whole.

The study found that there is a lack of understanding of these disorders amongst professionals. Improvement in education is required to ensure healthcare professionals are in possession of the correct information, regarding the different traits and symptoms of ASD and ADHD. This should include the gender differences that can be present.

Practice recommendations have also been identified. To guarantee that evaluations are carried out accurately and that all potential signs of these disorders may be easily identified, screening and diagnostic methods must be improved. Additionally, a quicker and more simple transition from referral to evaluation is needed, and people who are diagnosed must have access to support and treatment choices. The issues brought up in this study would also be lessened with better contact with service consumers.

These findings reinforce the need for improvement in communication, ongoing support, and education to ensure healthcare professionals are in possession of the correct information regarding the different traits and symptoms of ASD and ADHD, to include the gender differences that can be present, and to enhance patient health outcomes. When improvements are made in these areas, the availability of support and treatment will increase. Service users will have a more positive experience with their healthcare professionals, leading to better overall health outcomes. This approach promotes individualised, person-centred care, while proactively addressing and planning for an individual’s long-term needs.

Individualised person-centred care will be encouraged by changes in communication, knowledge, and service delivery. As a result, fewer checkups and possible hospital stays will result from anticipating a person’s long-term requirements. This will eventually ease some of the strain on the waiting lists for evaluations, treatments, and follow-up visits. 

Healthcare services will be better able to satisfy the needs of their clients if they offer person-centred care and treatment with better health outcomes. As a result, the healthcare provider’s standing and reputation will rise and the administration and provision of services will be improved.

## Figures and Tables

**Table 1:** Positive interactions between participants and healthcare professionals.

Participant Code	Quote
R4	My experience was an incredibly positive one, I was pleasantly surprised
R38	My GP was amazing and very helpful, she made me feel validated

**Table 2:** Negative interactions between participants and healthcare professionals.

Participant Code	Quote
R6	GP said you don’t look like you’ve got autism, occupational health consultant said the same
R21	GP couldn’t believe I might have ADHD because I have a degree! I felt so frustrated and quite angry with his attitude
R31	It was played down, and I was told I had survived this long what’s more time and they’d heard it all before
R40	I felt like I had to fight to be understood and taken seriously

**Table 3:** Participants’ comments regarding misdiagnosis.

Participant Code	Quote
R8	EUPD and cPTSD caused by the trauma of undiagnosed autism
R13	Anxiety and depression which I now understand has arisen from a lack of diagnosis. ADHD diagnosis has enabled me to begin therapy for these issues
R39	I was diagnosed borderline (BPD) for many years before and treated very badly, I actually have severe autism

**Table 4:** Participants’ experiences of differentiation of gender.

Participant Code	Quote
R33	It didn’t touch on my struggles as a woman and mother
R9	They dismiss women too easily. I had to go back with my husband and have him explain how it was appropriate
R40	It makes me really angry that my life has been impacted for all these years because I am female
R21	Many people, including healthcare professionals, will not be up to date with ADHD information and may apply gender bias
R25	If awareness of ADHD in women had been known earlier, it would have saved me a lot of traumatic experiences in life

**Table 5:** Participants’ comments regarding lack of support post-diagnosis.

Participant Code	Quote
R26	My childhood and current symptoms
R28	Accommodations, advocating for yourself
R38	Other health issues

**Table 6:** Participants responses regarding post-diagnosis discussion with healthcare professional.

Participant Code	Quote
R4	My GP never contacted me since my diagnosis, despite the report listing recommendations for support avenues
R40	They didn’t contact me I have had to constantly chase them for results and treatment

**Table 7:** Limited support options offered post-diagnosis.

Participant Code	Quote
R2	Given details of an organisation running 10-week courses for newly diagnosed adults
R6	Local charity helped. self-referral to government disability support scheme
R7	I was asked by the psychiatrist if I wanted talking therapy
R11	Medication and coaching support provided

**Table 8:** Timeframe from referral to assessment.

Participant Code	Quote
R9	Wait so far was 2 years, approximately another 2 years to wait
R25	Waiting times here are 5 years or more
R48	Waited 3 Years ADHD and 4 years for ASD
R49	Waited for NHS about 3 years then Right to choose for an additional 6 months
R17	Waited for 3 years and went private in the end
R28	Wait time was approximately12 months for autism, referred back to NHS for combined ADHD in writing in case want access to medication, could take total 8 years

**Table 9:** Participants in disagreement with diagnostic report.

Participant Code	Quote
R12	Diagnosed with Asperger’s syndrome which is a dx I am fundamentally opposed to. Dx inattentive ADHD when it should be combined
R22	They described it as mild and my experience of ADHD is not mild
R33	I didn’t like their interpretation of my behaviours; it didn’t mention the things I struggle with most

**Table 10:** Participants in agreement with diagnostic report.

Participant Code	Quote
R4	Even though I felt the outcome and diagnosis was accurate
R21	I’ve been diagnosed with combined type and that fits with symptoms as a child and growing up
R19	it did not say how it was currently impacting me, just how it had affected me in the past

**Table 11:** Participants’ comments regarding lack of support during assessment.

Participant Code	Quote
R26	The diagnosis was rushed, and the doctor was rude and dismissive of some of my concerns
R40	I was originally assessed by the NHS, the process was traumatic and took far too long, it was poorly executed with no explanation. I didn’t understand the questions, and I was never told the results despite months of chasing. I then paid for a private assessment

## Data Availability

All data generated or analysed during this study are included in this article, and additional data are available from the corresponding author upon reasonable request.
